# Genome-Wide Analysis of Aquaporins Gene Family in *Populus euphratica* and Its Expression Patterns in Response to Drought, Salt Stress, and Phytohormones

**DOI:** 10.3390/ijms251810185

**Published:** 2024-09-23

**Authors:** Boniface Ndayambaza, Jianhua Si, Dongmeng Zhou, Xue Bai, Bing Jia, Xiaohui He, Chunlin Wang, Jie Qin, Xinglin Zhu, Zijin Liu, Boyang Wang

**Affiliations:** 1 Key Laboratory of Ecohydrology of Inland River Basin, Northwest Institute of Eco-Environment and Resources, Chinese Academy of Sciences, Lanzhou 730000, China; ndayambazab78@mails.ucas.ac.cn (B.N.); zhoudongmeng@nieer.ac.cn (D.Z.); baixue18@yeah.net (X.B.); jiab@lzb.ac.cn (B.J.); hexiaohui@nieer.ac.cn (X.H.); wangchunlin@nieer.ac.cn (C.W.); qinjie18@lzb.ac.cn (J.Q.); zxinglin@yeah.net (X.Z.); liuzijin@nieer.ac.cn (Z.L.); wangboyang23@mails.ucas.ac.cn (B.W.); 2 University of Chinese Academy of Sciences, Beijing 100049, China

**Keywords:** aquaporins, water stress, abiotic stresses, gene expression, *P. euphratica*

## Abstract

Aquaporins (AQPs) play an essential role in membrane water transport during plant responses to water stresses centered on conventional upstream signals. Phytohormones (PHs) regulate plant growth and yield, working with transcription factors to help plants withstand environmental challenges and regulate physiological and chemical processes. The AQP gene family is important, so researchers have studied its function and regulatory system in numerous species. Yet, there is a critical gap the understanding of many of their molecular features, thus our full knowledge of AQPs is far-off. In this study, we undertook a broad examination of the AQP family gene in *Populus euphratica* via bioinformatics tools and analyzed the expression patterns of certain members in response to drought, salt, and hormone stress. A total of 22 AQP genes were examined in *P. euphratica*, and were categorized into four main groups, including TIPs, PIPs, SIPs, and NIPs based on phylogenetic analysis. Comparable exon–intron gene structures were found by gene structure examination, and similarities in motif number and pattern within the same subgroup was determined by motif analysis. The *PeuAQP* gene family has numerous duplications, and there is a distinct disparity in how the members of the *PeuAQP* family react to post-translational modifications. Abiotic stress and hormone responses may be mediated by AQPs, as indicated by the abundance of stress response elements found in 22 AQP genes, as revealed by the promoter’s cis-elements prediction. Expression pattern analysis reveals that selected six AQP genes from the PIP subgroup were all expressed in the leaves, stem, and roots with varying expression levels. Moreover, qRT-PCR analysis discovered that the majority of the selected AQP members were up- or down-regulated in response to hormone treatment and abiotic stress. Remarkably, *PeuAQP14* and *PeuAQP15* appeared to be highly responsive to drought stress and *PeuAQP15* exhibited a high response to salt stress. The foliar application of the phytohormones (SA, IAA, GA3, MeJA, and ABA) were found to either activate or inhibit *PeuAQP*, suggesting that they may mitigate the effects of water shortage of poplar water stress. The present work enhances our knowledge of the practical roles of AQPs in stress reactions and offers fundamental information for the AQP genes in poplar species. It also highlights a direction for producing new varieties of poplar species with drought, salt, and hormone tolerance and holds substantial scientific and ecological importance, offering a potential contribution to the conservation of poplar species in arid regions.

## 1. Introduction

Water is transported between the soil and the atmosphere by means of a system established by terrestrial plants [1]. Drawing water up to the leaf tissues is facilitated through a transpiration stream [2]. There are three distinct routes by which water moves through plant tissues: the transcellular pathway, which passes through cell membranes; the apoplastic pathway, which is exterior to the cells; and the symplastic pathway, which runs through the plasmodesmata that link cells [3,4]. Strict water transport and management in plants is indispensable for plant growth and development. Particularly significant in plant water transport are aquaporins (AQPs), which facilitate water transmembrane transport. Aquaporins were first found and studied in animals. The foundation for deciphering the molecular mechanisms underpinning water transport in plants was laid in 1993 when the first aquaporins, AtTIP1; 1 and the γ-tonoplast intrinsic protein (γ-TIP), were identified in *Arabidopsis* [5]. Major intrinsic proteins (MIPs) are a superfamily of highly conserved membrane proteins that includes aquaporins. Compared to animals and microbes, plants have a higher abundance and diversity of aquaporins [6]. Studying the molecular underpinnings of water transmembrane transport is made possible by the discovery of these aquaporins. Gaining more insight into the evolution of MIPs is made possible by the quick collection of genomic and transcriptome data from species that were previously unidentified. More substrates with particular physiological functions can be transported by plant aquaporins than by any other aquaporin since they are not only phylogenetically varied but also functionally advanced. Plant MIP superfamily proteins can be categorized into multiple subfamilies according to their structure and subcellular localization [7]. These successes offer new perspectives on the molecular underpinnings of plant water use and further the general advancement of the molecular depiction of plant membrane transport systems [8]. 

The quantity and action of aquaporins (AQPs) in cellular membranes modulate the transcellular water flow productivity, which in turn impacts numerous physiological functions [9,10]. AQPs are engaged in the transport of water to ease the channel of further minor solutes, like CO_2_, boric acid, H_2_O_2_, glycerol, urea, and silicic acid, through cell membranes [11]. Abiotic stressors such as salt, drought, and cold stress are made easier for plants to withstand but AQPs are also necessary to maintain water homeostasis and the hydraulic conductivity balance [12]. Additionally, it has been shown that AQPs are convoluted in numerous progressive procedures, as well as the closing of guard cells, petal movement, male fertility, regulation of stomata and petals, fruit ripening, and seed germination [13]. AQPs are characterized into seven subfamilies or subgroups to analyze the gene members of the cluster: tonoplast intrinsic proteins (TIPs), plasma membrane intrinsic proteins (PIPs), nodulin26-like intrinsic proteins (NIPs), small basic intrinsic proteins (SIPs), hybrid intrinsic proteins (HIPs), GlpF-like intrinsic proteins (GIPs), and uncategorized X intrinsic proteins (XIPs) [14]. A crucial class of transporters on the plasma membrane are plasma membrane aquaporins (PIPs) [15]. From the external location into the intracellular environment, they are the first to regulate the flow of water. Higher plants have large and diversified families of PIP proteins [8]. A total of 13 PIP homologs have been found in *A. thaliana* [16], 11 in rice (*O. sativa*), 15 in black cottonwood (*P. trichocarpa*), 28 in upland cotton (*G. hirsutum*), 22 in soybean (*G. max*), 14 in tomato (*S. lycopersicum*), 15 in rubber tree (*H. brasiliensis*), and 16 in flax (*L. usitatissimum*) [6]. PIPs are classified into two subgroups: PIP1 and PIP2. Phylogenetic investigations show that PIPs are found in bryophytes and gymnosperms, and that the PIP1 and PIP2 subgroups might be split apart before the monocot/dicot divergence [6]. Whereas PIP2 has a tinier N-terminus but a lengthier C-terminus, PIP1 has a lengthy N-terminus and a C-terminus that ends at the intracellular portion of transmembrane region 6 [17]. PIP1s and PIP2s are present throughout abiotic stresses like drought and salt in *A. thaliana*. Studies show that their expression decreases under salt treatment, leading to the slower recovery of hydraulic conductance and transpiration rates. The over-expression of *OsPIP1-1* or *OsPIP2-2* enhances tolerance to salt and drought [18]. Studies have shown that transgenic plants with increased stomatal conductance and leaf growth have PIP expression, which is linked to plant growth and organ formation [19]. 

Plants face real-time environmental changes and undesirable growth variations, negatively impacting economically important woody plants. Abiotic stresses like salinity, drought, and high temperatures lead to poor plant growth and reduction in forest tree yields [20]. Worldwide, abiotic stresses affect 19.5% (salinity) and 45% (drought) of agricultural land [21], with more severe abiotic stresses predicted in arid and semi-arid regions because of climate change [22]. Salinization and drying are believed to be the main reasons of the damaging of water and land resources, which restrict the productivity of woody plants globally and creates environmental issues, especially in arid regions. Salinity disrupts ion transport and homeostasis in the cells of plants by acting largely as an osmotic stressor. In addition, it degrades proteins and membranes, induces oxidative stress, and triggers signaling cascades that alter gene expression. Salt tolerance is a complex feature that is organized by several genes and includes a number of physiological and biochemical processes. Drought stress impacts plant homeostasis, gas exchange, seed development, and water processes. Plants have advanced defenses that include closing the stomata, lowering transpiration, generating ABA, and storing H_2_O_2_ [23]. Family genes play a crucial part in plant reactions. Scientific research has focused on finding genes involved in drought and salt stress response and on understanding systems underlying drought and salt tolerance [24]. 

Woody plants, like *P. euphratica*, are known for their drought and salt tolerance, making them valuable components of riparian ecosystems in arid regions [25]. This non-halophyte and mesophyte species is often used as model forest trees because of their ease of regeneration and ecological relevance. *P. euphratica’*s unique leaf heteroblasty pattern contributes to its resilience to drought and other environmental stresses [26]. There are no reports on *P. euphratica* despite extensive research on the characterization and function of the AQP family gene in many other plants. This work is the first to give a comprehensive analysis of the AQP (called *PeuAQP*, PIPs) genes in *P. euphratica* via large-scale transcriptome data from the NCBI database and a recently released genome database. All nonredundant sets of *PeuAQP* genes were found in this study, and we then examined the data using phylogenetic relationships, chromosome locations, motif analysis, gene duplication, gene structures, cis-acting elements, subcellular prediction, phosphorylation site analysis, 3D modeling prediction, collinearity, and protein–protein interaction prediction of 22 novel AQP genes in *P. euphratica*. Additionally, we used quantitative real-time PCR (qRT-PCR) to analyze the expression of the six selected *PeuAQP* genes under different abiotic stress treatments, like salt, hormones, and drought. The acquired data clearly represent an extremely valuable resource for upcoming comprehensive research on the advancement *PeuAQP* (PIPs) functions, which will unquestionably be highly beneficial in the upcoming probe of *P. euphratica* stress-related responses. These discoveries will also offer a foundation and genetic resources for breeding species with high stress tolerance, allowing for the focused breeding of improved genes to increase poplar survival and productivity in arid areas.

## 2. Results

### 2.1. Comprehensive Analysis of AQPs Genes in P. euphratica

**Figure 1:**
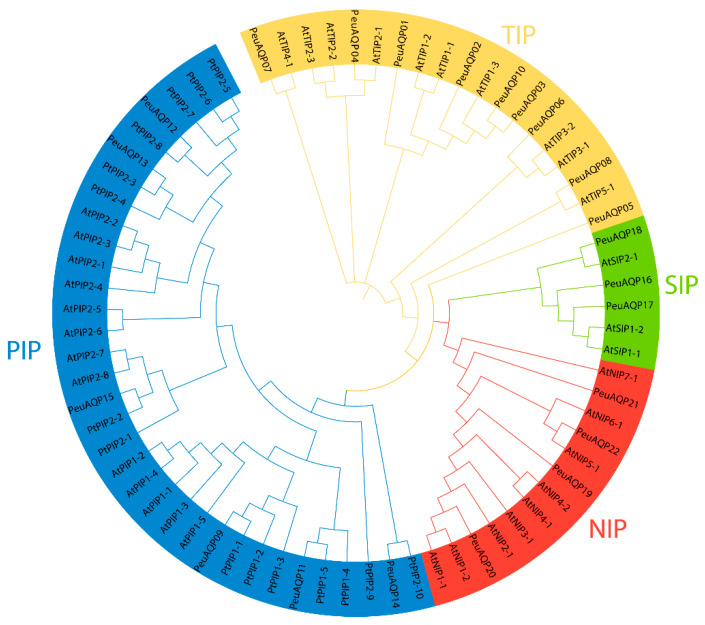
Phylogenetic tree analysis of AQP proteins in *P. euphratica* and two other species. The full length of the amino acid sequences of twenty-two PeuAQP proteins, fifteen PtAQP proteins, and thirty-five AtAQP proteins were aligned via Clustal W; a phylogenetic tree was created through MEGA 7 by the neighbor-joining (NJ) method with 1000 bootstrap replicates and was visually enhanced by categorizing it into four subfamilies. Then, we categorized each subfamily by color and further labeled them.

Using the keyword “aquaporin or MIP”, a total of 56 gene sequences were acquired from the NCBI databank (www.ncbi.nlm.nih.gov/protein, accessed on 19 April 2024). Of these, 22 AQP genes (PeuAQPs) had two NPA motifs and one complete MIP domain (Table S1). Sequence cluster studies of *P. trichocarpa*, *P. euphratica*, and *A. thaliana* led to the classification of those *PeuAQPs* into four subfamilies: six PIPs, nine TIPs, three SIPs, and four NIPs (Figure 1). The PIP1 and PIP2 subgroups, consisting of one PIP1 and four PIP2s, were part of the PIP subfamily. Three TIP1s, one TIP2, two TIP3s, one TIP4, and one TIP5 made up the five subgroups that comprised the TIPs subfamily. There were two subgroups in the SIPs subfamily: SIP1 and SIP2. There were three subgroups in the NIPs subfamily: two NIP1s, one NIP2, and one NIP5. PeuAQPs were named according to their chronological order in the NCBI protein database after removing redundant proteins. Amongst the 22 predicted PeuAQP proteins, the main protein was *PeuNIP5;1*, with 300 amino acids (Aas), whereas the least protein was *PeuNIP1;1*, with 196 Aas with an average length of 261.6 (Supplementary Materials Table S1a). The Zhang et al. protein database contained 50 potential AQP protein sequences, the longest of which ranged from 531 to 183 Aas (Supplementary Materials Table S1b). These fifty AQP protein sequences were discovered in Zhang et al.’s protein database, sixteen of which matched those reported by Ma et al. (Supplementary Materials Table S1a) as these proteins shared the same identical protein numbers. However, their sequence numbers were different to each other. There were three subgroups, including TIPs, PIPs, and NIPs, found in these fifty proteins reported by Zhang et al.; nevertheless, there were no SIP subgroups present in these protein sequences, as Ma et al. reported (as illustrated in Supplementary Materials Table S1a). We were able to identify 22 AQP proteins in *P. euphratica* by combining two protein databases, including these SIP subgroups, and renamed them whilst rendering gene ID on NCBI.

### 2.2. Gene Structure and Motif, Gene Mapping of Chromosome, and Phosphorylation Site Proteins Analyses of PeuAQPs

**Figure 2:**
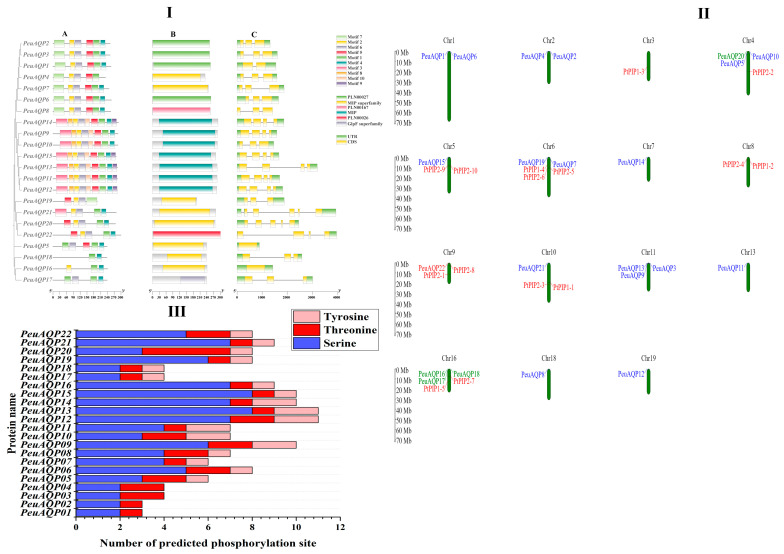
(**I**) Phylogenetic relationship, motif composition, domain, and gene structure of AQP genes in *P. euphratica*. (**A**) The multiple positions of full-length AQP protein sequences from three species were acquired via Clustal W, and a phylogenetic tree was built with MEGA 7 by the neighbor-joining (NJ) method with 1000 bootstrap replicates. The depiction image of conserved motifs was acquired using motif elicitation (MEME) in AQP proteins. The 10-motif composition model in the PeuAQP whole amino acid sequences was primed by MEME.XML through TBtools software (Toolbox for Biologists v1.130, University of Science and Technology, Wuhan, China). Various motifs are represented by boxes of diverse colors. (**B**) Domain of AQP gene family analysis. (**C**) Exon/intron structure of the AQP genes. Green and yellow boxes indicate exons and gray lines represent introns of the AQP genes. (**II**) Chromosomal locations of AQP genes in poplars species. Twenty-two *PeuAQP* genes were found across chromosomes 1, 2, 3, 4, 5, 6, 7, 8, 9, 10, 11, 13, 16, 18, and 19. The corresponding chromosome numbers are shown at the uppermost section of every chromosome, respectively. (**III**) Depiction figure of predicted phosphorylation sites of PeuAQP proteins. Network analysis was carried out on serine, threonine, or tyrosine residues. Each network pocket site of same prediction was observed in each subfamily.

Exon–intron structures are essential to the evolution of plants. *PeuAQPs’* exon–intron design and conserved motifs were examined. There were four exons in most PIP subfamilies. With the exception of TIP1;1 and TIP3;1, which have two introns and one exon, all TIP subfamilies had three exons. With the exception of SIP1;1, which had one intron, all SIP subfamilies had three exons. There were five exons in NIP subfamilies, except NIP1;1 and NIP5;1 which had three and four exons, respectively (Figure 2I(C)). All PeuAQPs, with the exception of TIP1;3 which does not have Motif 3, contained Motifs 1 and 3, as illustrated in Figure 2I(A). The presence of seven motifs in the PIP and TIP subfamilies indicates that these motifs were strongly conserved in *P. euphratica*. Only the PIP subfamilies possessed motif 5 and motif 9. The 22 *PeuAQP* genes were unevenly dispersed amongst 19 chromosomes and demonstrated the gene loci information of AQP gene family members in *P. trichocarpa* [6]. *PeuAQPs* were largely dispersed at both ends of chromosomes, and the density of *PeuAQPs* diverse on distinct chromosomes. Most of the *PeuAQPs* were positioned on Chr16 (three genes, 13.63%); Chr 11 (three genes, 13.63%); Chr 4 (three genes, 13.63%); Chr 1 (two genes, 9.09%); Chr 2 (two genes, 9.09%); Chr 6 (two genes, 9.09%); and Chr 5, Chr 7, Chr 10, Chr13, Chr18, and Chr 19 (one gene; 4.54%). However, there were no *PeuAQPs* members on Chr 8, Chr 9, Chr 12, Chr14, Chr15, and Chr 17 (Figure 2II). Five paralogous pairings were found in the *PeuAQP* family established in the evolutionary interactions, gene structures, and motifs of the *PeuAQPs* (Supplementary Materials Table S1a). Consequently, the four pairs (*PeuAQP9*/*PeuAQP10*, *PeuAQP11*/*PeuAQP12*, *PeuAQP16*/*PeuAQP17*, and *PeuAQP20*/*PeuAQP22*) were produced by whole-genome duplication, and one paralogous pair (*PeuAQP2*/*PeuAQP3*) may have resulted from a tandem duplication event. Only the estimated divergence times of three of the five pairings (*PeuAQP11*/*PeuAQP12*, *PeuAQP16*/*PeuAQP17*, and *PeuAQP20*/*PeuAQP22*) generated and determined by 11 and 20 MYA and Ka/Ks ratios. However, due to the high divergence value (ps ≥ 0.75), the *PeuAQP20*/*PeuAQP22* Ka/Ks ratio was not produced. For each of the three paralogous pairs, the substitution rate ratios between nonsynonymous (Ka) and synonymous (Ks) ratios were determined (Table 1). Every Ka/Ks ratio was much lower than 1.

One popular technique for altering proteins is phosphorylation, which has an impact on the interactions, stability, subcellular location, structure, and activity of proteins [27]. The reversible phosphorylation of proteins, primarily occurring on serine, threonine, or tyrosine residues, is a highly significant and extensively researched post-translational modification. It is essential for controlling extensive biological functions, like cell cycle, proliferation, apoptosis, and signal transduction pathways. This analysis identified the phosphorylation of the *PeuAQP* family gene, and the results revealed a total of 62 phosphorylation sites, including serine, threonine, and tyrosine, which were dispersed extensively throughout all *PeuAQP* sequences (Figure 2III). Of these locations, serine had the highest number of phosphorylation sites, namely 99; followed by phosphorylated threonine sites, at 34; and phosphorylated tyrosine sites, at 24. Our results analysis of all phosphorylation sites revealed that *PeuAQP13* and *PeuAQP15* had the highest number of complete serine phosphorylation sites (8), followed by tyrosine phosphorylation sites (2), and threonine phosphorylation sites (1) (Supplementary Materials Table S3).

### 2.3. PeuAQPs Synteny Analysis

**Figure 3:**
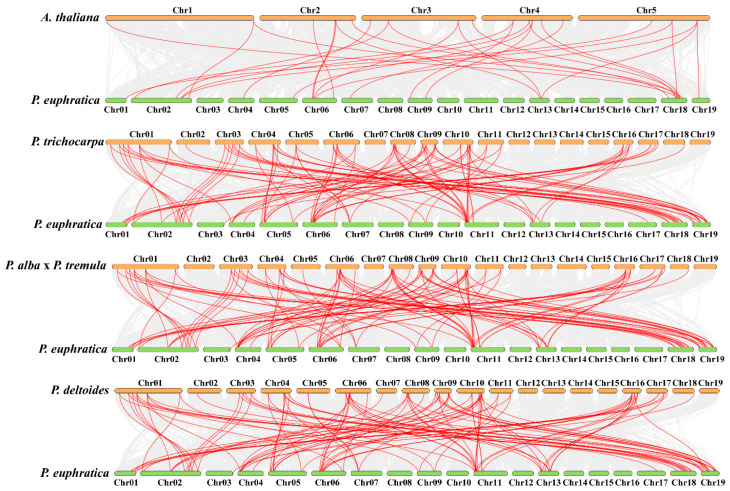
Comparative genomics analysis of AQP gene collinearity amongst *P. euphratica* and four demonstrative plants (*A. thaliana*, *P. trichocarpa*, *P.alba x P. Tremula*, and *P. deltoides*). The gray lines in the background designate blocks of collinearity inside *P. euphratica* and the designated plants, while the red lines specify homozygous AQP gene pairs.

To learn more about the evolutionary developments of *PeuAQPs* in *Populus* through the comparative genomics, a synteny analysis of AQPs amongst *P. euphratica* and the other four species (*A. thaliana*, *P. trichocarpa*, *P. deltoides WV94-445_v2.0*, and *P. alba* × *P. tremule\Populusalba HAP1_717_v5.0*) was carried out: twenty-eight pairs with *P. euphratica* and *A. thaliana*; ninety-five pairs with *P. trichocarpa* and *P. euphratica*; one hundred and three pairs with *P. euphratica* and *P. deltoides WV94-445_v2.0*; and lastly, one hundred and three pairs with *P. euphratica* and *P. alba* × *P. tremule*\*Populusalba HAP1_717_v5.0*. The *P. trichocarpa* was among the AQP synteny segments found amongst *P. euphratica* and the other species (Figure 3 and Supplementary Table S2). This finding showed that AQP synteny within the poplar species was more limited than AQP synteny in *P. euphratica* and *A. thaliana*. These findings proposed that the AQP gene family in Salicaceae is largely preserved, neither dramatically expanding nor contracting. In particular, these two poplar species showed the high synteny associations of pairs within *PeuAQP* genes, suggesting that these AQP had synteny sections that probably predate the original separation. The Salicaceae whole genome duplication may have enabled the preservation of AQP-included synteny segments. We found that *PeuAQP15* (populus_peu08687.t1), *PeuAQP14* (populus_peu14092.t4), and *PeuAQP8* (populus_peu34202.t1) were syntenic with three gene pairs in *A. thaliana*, while the *P. trichocarpa*, *P.alba* × *P.tremule*\*PopulusalbaHAP1_717_v5.0*, and *P. deltoidesWV94-445_v2.0* gene pairs had a higher number of AQP members of the synteny. The *PeuAQP* genes proved a significant degree of orthology with the reference genomes, with *P. trichocarpa* establishing a momentous 43-degree orthology level with the reference genomes, which were distributed across chromosomes 1, 2, 3, 4, 5, 6, 7, 9, 11, 13, 17, 18, and 19 of *P. euphratica*, and *A. thaliana*, with 19 orthologous gene pairs on the chromosomes 1, 2, 4, 5, 6, 7, 9, 13, 18, and 19 (Figure 2II and Supplementary Materials Table S2) of *P. euphratica*. Most *PeuAQP* members could not be linked with any syntenic gene pairs of *A. thaliana*’ chromosomes.

### 2.4. The 3D Modeling Prediction and Network Analysis of PeuAQP Genes

**Figure 4:**
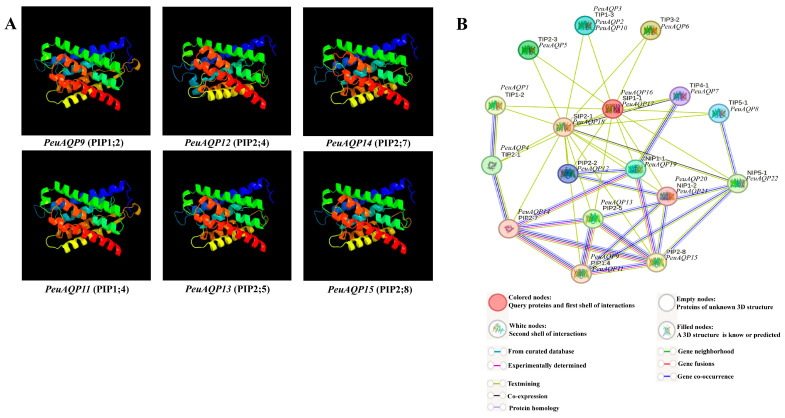
(**A**) The 3D structure analysis of the selected six *PeuAQP* genes in the subfamily of PIP. Modeled at a 100% confidence level by using Phyre2 server and the percentage residues ranged from 78 to 98%. (**B**) Schematic reveals the protein interaction network of PeuAQPs. Every node embodies a protein, and every edge characterizes an interaction.

To better comprehend the AQP gene family in *P. euphratica*, we were motivated by prior research that has shown this family gene has a sizable subfamily associated with abiotic stress, which includes plasma membrane intrinsic proteins (PIPs) in several plant species [28,29,30]. Plasma membranes are the localization site for PIPs, a subfamily of aquaporins that facilitates rapid and regulated water transfer across membranes [30]. In this work, we identified four categories of the AQP family gene (TIPs, PIPs, SIPs, and NIPs), and PIPs were selected for this genome-wide analysis. The substrate specificity and solute penetration proportion of AQP genes through the usage of 3D structure proteins of plant AQPs can aid in improving our comprehension of PeuAQPs’ ability to withstand abiotic stresses [31]. We used the Phyre2 server display to predict the structural features of all six PeuAQPs selected in homology-based tertiary (3D) structure protein models. We employed a computational online tool, through which hidden Markov model proteins were aligned using HMM-HMM [32], and used a 100% confidence interval for 3D protein modeling. Based on the aforementioned 3D modeling studies, the vibrant shape and stability of these particular proteins could play a momentous role in their response to abiotic stresses. The most significant issues in computational structural biology are related to homological 3D protein structures [33]. Every 3D protein model was built with a 100% confidence interval, and the range of residue coverage was from 78 to 98% (Figure 4A). A conserved hourglass-like structure made up of two short helices (HE and HB) [34] and one to six TM helices (H1 to H6) were present in the PeuAQP 3D protein model. In the middle of the membrane, near to one another, were loops HE and HB. The boron (B) and silicon (Si) substrate choosiness of the PeuAQPs was demonstrated by these anticipated 3D models, which also served as a crucial foundation for the protein efficient investigation of the AQP genes of *P. euphratica*. 

A protein–protein interaction network search of all discovered genes was carried out on each subfamily, where these proteins were highly significant, in order to understand the importance of the six targeted *PeuAQPs* during protein interactions. Building an inclusive regulatory network of a subset of species (*P. euphratica* versus *A. thaliana*) was our goal, and we focused on the predictions of *PeuAQPs* links to abiotic stressors. It can be seen that the *PeuAQP* gene family has a contiguous mutual relationship network with the *PtrAQP* genes within it and bears a significant resemblance to the genes’ roles in *A. thaliana* that were used as a reference [35]. This reveals a high probability that the structures and activities of the corresponding genes are the same. Numerous interaction patterns, including one-to-one, one-to-many, and many-to-many, were identified within this network. To learn more about AQP-dependent regulatory interactions under abiotic stressors, we separated out several sub-networks from the previously mentioned regulatory network. Rendering to our research, the AQP gene family is important because it ought to function as a water channel that could help poplar species move water transversely through their cell membranes. The essential network elements were analyzed, such as PIP 2;8 and PIP 2;7 in *A. thaliana* (see Figure 4B). This subgroup of aquaporin PIP could play a crucial role that could be connected to the osmoregulation in plants under high osmotic stress, like high salt conditions. In this work, these two interesting subgroups shared network interactions with *PeuAQP14* and *PeuAQP15*. In contrast, *PeuAQP10* shared an interaction network with the TIP subgroup, which is also found in plants and plays a critical role in osmoregulation in situations involving significant osmotic stress, including high salinity. Second, PIP2;2 in *A. thaliana*, which interacts with *PeuAQP11*, *PeuAQP12*, and *PeuAQP13*, revealed that in circumstances of decreased transpiration, this subgroup could be crucial to the processes of root water intake and osmotic fluid transport. It was observed that their utility is moderated by Hg(2+) and inhibited by cytosolic acidosis, which happens throughout anoxia in roots [36]. These associations support the phylogenetic relationships and clearly demonstrated the selectivity and affinity of the positive proteins, which are more resilient to plant stress.

### 2.5. Cis-Acting Elements in the Promoter Regions of PeuAQP Genes Analysis

**Figure 5:**
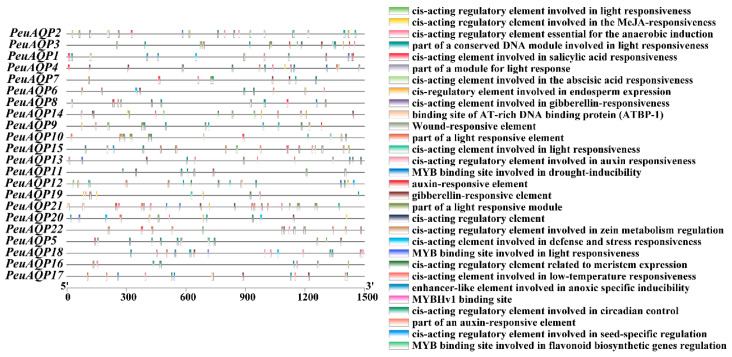
Cis-acting elements in the promoter region of the *PeuAQP* gene family. Distinct colored boxes denote diverse cis-elements.

To explore the probable role of the *PeuAQP* genes in the abiotic stress response, the 1500 bp promoter sequences of the gene were employed, and its cis-elements were examined via the PlantCARE database (Figure 5). The promoter region of the PeuAQP gene had a total of 30 cis-acting elements linked to plant hormone and stress responses, including components associated with growth and development, stress, and light. Drought stress, injury stress, and hypoxia stress response elements were among the cis-acting components associated with the stress response. Our results reveal that abiotic stresses are present in nearly all of the poplar AQP family genes in terms of cis-acting regions responsible for the abiotic stress response. Furthermore, the AQP genes of *P. euphratica* contained the MYC and MYB (*PeuAQP9*, *PeuAQP13*, *PeuAQP14*, *PeuAQP12*, *PeuAQP6*, *PeuAQP15*, *PeuAQP11*, *PeuAQ2*, *PeuAQP4*, *PeuAQP16*, and *PeuAQP1*) promoters, which caused abiotic stress in a variety of species, including salt and drought stress. The first category included elements that responded to PHs, including the ABRE, TGACG, P-box, TATC, and GARE molecules. The CGTCA-motif and TGACG-motif were the cis-acting regulatory elements engaged in responses to methyl jasmonic acid (MeJA). The TCA-element was the cis-acting regulatory element implicated in salicylic acid (SA) responses. In the plant defense signaling, SA and MeJA were both important players [37,38]. Thus, pathogen resistance may be influenced by certain AQP genes. ABRE was one of the cis-acting regulatory elements implicated in abscisic acid (ABA) responses. The regulatory components associated with gibberellin (GA) comprised GARE-motif, TATC-box, and P-box. The TGA-element and AuxRR-core were two examples of cis-acting regulatory elements that responded to auxin (IAA). Since ABA, GA, and IAA may regulate the expression of some AQP genes, they play important roles in the growth and survival of plants (Figure 5). Abiotic and biotic stress responsive elements comprised the second type of cis-elements; these included elements that were induced by anaerobic treatment (ARE), were anoxia-specific inducible (GC-motif), were drought-inducible (MBS, *PeuAQP17*, *PeuAQP13*, *PeuAQP18*, *PeuAQP15*, *PeuAQP2*, *PeuAQP4*, *PeuAQP1*), and were defense and stress responsive (TC-rich repeats, *PeuAQP18*, *PeuAQP6*, *PeuAQP1*, *PeuAQP20*) (Figure 5). The AQP gene family also contained additional PHs-sensitive (like ERE) and abiotic and biotic stress-responsive (like wounds, *PeuAQP22*, and low temperature, *PeuAQP17*, *PeuAQP21*, *PeuAQP18*, *PeuAQP6*, *PeuAQP15*, *PeuAQP1*) elements (Figure 5). Plant growth and development elements comprised the third class. These included the highly represented endosperm expression cis-acting regulatory elements (GCN4_motif), a cis-acting regulatory element linked to meristem expression (CAT-box), and circadian control cis-acting elements (circadian), which were found in the *PeuAQP* genes. Among the light responsive elements in the final class were the (G-box), (AE-Box), (ACE), and (GT1-motif) (Table 2 and Supplementary Materials Table S4).

### 2.6. Responses of AQPs to Exogenous of Various Hormones in P. euphratica Leaves

**Figure 6:**
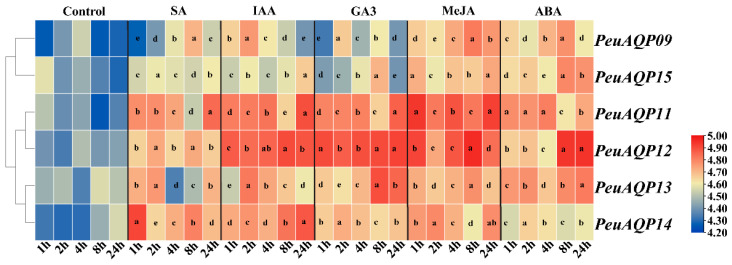
The relative expression analysis of the *PeuAQP* genes in response to SA, IAA, GA3, MeJA, and ABA treatments after 1 h, 2 h, 4 h, 8 h, and 24 h. They were conducted for the time (h) of each treatment. The heat map figure was based on the calculation of triplicates of qRT-PCR data and the transformation of the log2 algorithm. The letters a, b, c, d and e denote significant differences, and the least significant differences (LSDs) test was used to calculate the *p*-values (*p* ≤ 0.05).

The expression levels of candidate genes were examined in order to investigate how AQP genes respond to exogenous hormones in *P. euphratica* leaves (Figure 6). Following the administration of the stress hormone salicylic acid (SA), there was a down-regulation of *PeuAQP9* (PIP1;2) and *PeuAQP15* (PIP2;7) expression levels; an initial down- and subsequent up-regulation of *PeuAQP11* (PIP1;4), *PeuAQP12* (PIP2;4), and *PeuAQP14* (PIP2;7) expression levels; and an up-regulation of *PeuAQP14* (PIP2;7) expression levels. Moreover, after auxin (IAA) application, the expression levels were significantly up-regulated in *PeuAQP11* (PIP1;4), *PeuAQP12* (PIP2;4), *PeuAQP13* (PIP2;5), and *PeuAQP14* (PIP2;7). Next, *PeuAQP9* (PIP1;2) and *PeuAQP15* (PIP2;8) were down-regulated in *P. euphratica* leaves following gibberellin (GA3) treatment, whereas the expression levels of *PeuAQP11* (PIP1;4), *PeuAQP12* (PIP2;4), *PeuAQP13* (PIP2;5), and *PeuAQP14* (PIP2;7) were up-regulated. After methyl jasmonate (MeJA) application, the expression levels of *PeuAQP* genes in *P. euphratica* leaves were abruptly up-regulated in *PeuAQP9* (PIP1;2), *PeuAQP15* (PIP2;8), *PeuAQP11* (PIP1;4), *PeuAQP12* (PIP2;4), *PeuAQP13* (PIP2;5), and *PeuAQP14* (PIP2;7). Lastly, the expressions of abscisic acid (ABA) *PeuAQP9* (PIP1;2), *PeuAQP15* (PIP2;8), *PeuAQP11* (PIP1;4), *PeuAQP12* (PIP2;4), *PeuAQP13* (PIP2;5), and *PeuAQP14* (PIP2;7) were induced and sharply up-regulated. 

### 2.7. Response and Expression Pattern of AQPs to Drought Stress in Populus Tissues/Organs

**Figure 7:**
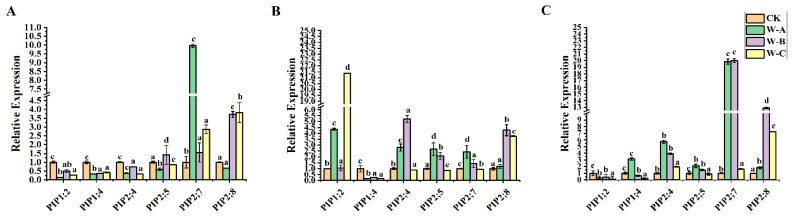
The expression analysis of *PeuAQP* genes in response to drought stress. Expression analysis of *PeuAQPs* in various tissues including (**A**) leaves, (**B**) stem, and (**C**) roots. The W-A (5 days), W-B (10 days), and W-C (20 days), respectively, represent four drought treatments with various soil volumetric water contents (soil-VWC): control (41 ± 1% soil-VWC), mild drought (31 ± 1% soil-VWC), moderate drought (21 ± 1% soil-VWC), and severe drought (11 ± 1% soil-VWC). Relative expression in the control was set 1. The *PeuAQPs* relative expression were analyzed vs. control (Duncan’s test and one-way ANOVA). Data were presented as mean ± standard error (SE). Error bars symbolize the standard deviations of three replicates. The letters a, b, c, and d denote significant differences, and the least significant differences (LSDs) test was used to calculate the *p*-values (*p* ≤ 0.05). Three biological repeats were used in this study.

Next, we used qRT-PCR analysis to perform gene expression study across various *P. euphratica* organs in order to identify potential down- and up-regulated genes. In order to further explore whether *PeuAQPs* respond to abiotic stress, we explored the expression of AQP in *P. euphratica* leaves, stems, and roots under four different periods of drought stress (control, mild drought, moderate drought, and severe drought stress). As presented in Figure 7C, the relative expression levels of *PeuAQP12* (PIP2;4), *PeuAQP13* (PIP2;5), *PeuAQP14* (PIP2;7), and *PeuAQP15* (PIP2;8) genes in roots were up-regulated in the four different periods of drought water stress, and the responses in the mild to moderate drought periods were the most obvious. This was followed by severe drought stress, which was the most sensitive, and all genes showed a peak value after 10 days of treatment. *PeuAQP14* (PIP2;7), *PeuAQP13* (PIP2;5), and *PeuAQP15* (PIP2;8) were less sensitive to osmotic stress than other genes, and their expression levels changed little in these three tissues (Figure 7). Except for *PeuAQP11* (PIP1;4) and *PeuAQP9* (PIP1;2), all genes showed the significant trend of up-regulation in leaves and roots in 10 days and then decrease in response to drought stress at 20 days (Figure 7A). Consequently, apart from *PeuAQP11* (PIP1;4) which was down-regulated in the stems, other AQP genes, including *PeuAQP9* (PIP1;2), *PeuAQP12* (PIP2;4), *PeuAQP13* (PIP2;5), *PeuAQP14* (PIP2;7), and *PeuAQP15* (PIP2;8), were significantly elevated for 10 days after treatments. Interestingly, *PeuAQP15* (PIP2;8) showed a continuously high expression for 20 days (Figure 7B). It is speculated that AQPs may play a progressive regulatory function in the tolerance of *P. euphratica* to drought stress. AQPs were highly expressed in all *P. euphratica* organs; however, the examination of drought stress revealed that the uppermost absolute expression originated in the leaves and roots (Figure 7). As a result, we only gave consideration to candidates exhibiting strong AQP expression that was higher in the stems.

### 2.8. Response and Expression Pattern of P. euphratica AQP Family Genes under Salt Stress

**Figure 8:**
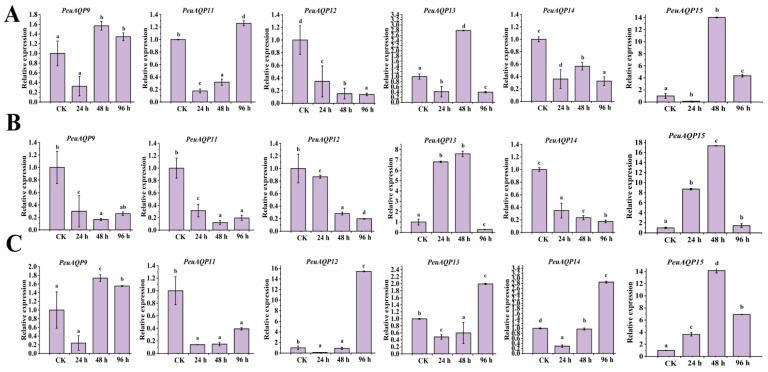
The qRT-PCR analysis of the six selected *PeuAQP* genes in response to NaCl treatment. Expression analyses of *PeuAQPs* in different tissues including (**A**) leaves, (**B**) stem, and (**C**) roots. Relative expression in the control was set 1. The *PeuAQPs* relative expression vs. control was analyzed (Duncan’s test and one-way ANOVA). Data were presented as mean ± standard error (SE). Error bars symbolize the standard deviations of three replicates. The letters a, b, c, and d denote significant differences, and the least significant differences (LSDs) test was used to calculate the *p*-values (*p* ≤ 0.05). Three biological repeats were used in this study.

We also examined the expression patterns of six AQP gene family members under salt stress in order to understand the *PeuAQP* gene’s response to abiotic stress. The results in Figure 8 illustrate show that six *PeuAQP* genes exhibit variable degrees of salt stress response, and *PeuAQP9* (PIP1;2), *PeuAQP13* (PIP2;5), and *PeuAQP15* (PIP2;8) expression was up-regulated after 48 h of NaCl treatment in leaves (Figure 8A). *PeuAQP13* (PIP2;5) and *PeuAQP15* (PIP2;8) expression was up-regulated and peaked after 24 h and 48 h of NaCl treatment but decreased at 96 h of NaCl treatment in stems (Figure 8B). Whereas the relative expression levels of NaCl in stems, *PeuAQP13* (PIP2;5), and *PeuAQP15* (PIP2;8) were markedly elevated, it peaked after 24 h and 48 h, and then declined after 96 h (Figure 8B). After 96 h of NaCl in roots, *PeuAQP12* (PIP2;4), *PeuAQP13* (PIP2;5), and *PeuAQP14* (PIP2;7) showed a notable upregulation and peaked (Figure 8C). On the other hand, the expression levels of other AQP genes varied significantly, whereas *PeuAQP12* (PIP2;4) was down-regulated in leaves, and *PeuAQP12* (PIP2;4) and *PeuAQP14* (PIP2;7) were down-regulated in stems. Furthermore, *PeuAQP15* (PIP2;8) genes were up-regulated after 48 h and 96 h of NaCl treatment in roots, demonstrating that some AQP genes varied in the response of *P. euphratica* to salt stress. A cluster analysis discovered that the responses to the NaCl stimulation of *PeuAQP* genes belonging to the same evolutionary branch are quite comparable. These findings imply that the *PeuAQP13* (PIP2;5) and *PeuAQP15* (PIP2;8) genes may interact in response to NaCl stress, even if they may have distinct roles and functions at various periods. Both of these genes may be important in the response to NaCl stress. 

## 3. Discussion

A collective numeral of plant genomes were well assembled thanks to the quick advancement of genome sequencing technology, making it easier to identify a variety of plant genomes. The *P. euphratica* genome has been sequenced and made available for public use on the NCBI database [39,40]. This makes it easier to study the P. euphratica gene family and its gene evolution. Plant life is based on water, and water molecules can participate in various aspects of life by entering the cell through the cell membrane and entering the environment. Seventy percent of transmembrane transport is carried out by particular membrane proteins called AQP proteins, and the AQP genes that regulate plant growth and development are numerous, diverse, and widely distributed inside plant cells [41]. Because these proteins regulate the absorptivity of membranes to water, they are indispensable for adaptation to harsh environmental conditions. In addition, aquaporins facilitate water transport during tissue expansion and are important in the water balance of leaves [42]. Plant AQP proteins belong to the MIP superfamily of intrinsic membrane proteins. They take part in numerous procedures, like cellular osmoregulation, lateral root formation, seed germination, carbon fixation, nutrient uptake, response to abiotic stress, and hormone signaling [43]. Plant genomes contain AQPs in large quantities, and reports of genome-wide AQP analysis in a range of higher plants have been made. Numerous plants have been the subject of wide research into the biological aspects and bioinformatics analysis of AQP genes [44]. Nevertheless, no research has been carried out on the functional assessments of the *P. euphratica* AQP family [6]. Thus, it is crucial to perform a genome-wide analysis of AQPs in *P. euphratica*, an economic forest tree species for arid regions. In the present study, 22 candidate genes were acknowledged in *P. euphratica* and the expression patterns of these genes were analyzed. 

A phylogenetic tree was created with AQPs from *P. euphratica*, *A. thaliana*, and *P. trichocarpa* in order to understand evolutionary links and the ability of PeuAQPs to withstand to abiotic stress and PHs, as illustrated in Figure 1. The total number of AQPs in this study compared to those in *E. guineensis*, *V. vinifera*, *O. sativa* [44], *A. thaliana*, and *R. communis* [45] were lower than thirty of these genes in *E. guineensis* and *V. vinifera*. PIPs and TIPs were altered in number amongst the two genomes compared to the other subfamilies. PIPs and TIPs have been recounted to consist of 12 and 13 members in *C. cajan*, respectively, whilst *P. euphratica* has been identify as consisting of six PIPs, nine TIPs, three SIPs, and four NIPs. PeuTIPs are divided into five subgroups, TIP1, TIP2, TIP3, TIP4, and TIP5, and this grouping is consistent with those previously identified in species such as *H. vulgare* cv Sahara [46] and *B. vulgaris* [47]. Two subgroups of *PeuPIPs*, PIP1 and PIP2 are identified. Subsequently, *PeuSIPs* are categorized into two subgroups, SIP1 and SIP2, while *PeuNIPs* are divided into three subgroups (Supplementary Materials Table S1). These divisions align with the AQP description groups that have been previously examined by scholars [48]. This shows how diverse these genes are among species, and it is postulated that throughout the progression of plant advancement, AQP gene duplication and functional differentiation took place. 

Many *PeuAQP* candidates had comparable traits with *P. trichocarpa*, but only a small number of these two poplar species’ consistent homologous genes were identified in this poplar species. This might be as a result of *P. euphratica’*s genome not being as thoroughly sequenced as *P. trichocarpa’*s. It is also feasible that these additional members evolved in *P. trichocarpa* following the split of the two species or that *P. euphratica* lost these ancestral sequences [49]. The subcellular location of aquaporins (AQPs) demonstrates variability because plant cells are highly segregated. Plants have AQPs in every cellular and subcellular compartment. While the NIP subfamily member NIP5;1 in *A. thaliana* shared the same position for the plasma membrane, different subcellular localizations were detected in the AQP members of *A. thaliana* [50]. Only the plasma membrane is shared by the *PeuAQP* members in these four subgroups: TIPs, PIPs, SIPs, and NIPs.

We examined the chromosomal location, gene structures, expression patterns, conserved motifs, exon–intron distribution, and evolutionary relationships of *PeuAQPs*. Certain duplication gene pairs were preserved in *P. trichocarpa* and *P. euphratica* despite the exponential decline in the number of resulting duplicated genes [51]. Previous research reveals that PIP genes in *A. thaliana* and *Populus* have a comparable exon–intron organization, with most *PtPIP* and *AtPIP* genes having three introns [51,52], conversely *OsPIP* genes contain zero to four introns. This suggests that PIP genes digress amongst monocots and dicots, and their expression is strongly controlled by their promoter activity. Protein sequences remain preserved amongst *PtPIP1* and *PtPIP2*, except for NPA motifs [53]. PIP2 proteins have a greater water absorbency and a smaller N-terminal region [54]. The phosphorylation of plant AQPs is crucial for fast channel gating, protein transferring, and pore opening and closing. Loop B and N- and C-terminal tails represent vital sites for water channel transportation. The phosphorylation of serine residue can occur both in vivo and in vitro [55]. On the other hand, the phosphorylated serine biosynthesis pathway connects nitrogen metabolism and results in plant development [56]. It also plays a momentous role in responding to abiotic stress in plants [57]. Our analyses show that the PIP subgroup is highly expressed in *PeuAQP* members compared to other subgroups (as illustrated in Supplementary Materials Table S3 and Figure 2III). Our findings suggest that the PIP subgroup has more significant sites for water channel regulation in the plasma membrane. In this study, the expression patterns of genes suggest that their location has a strong relationship to their function, with PIPs being more frequent in roots than in leaves. The utility of PIP subgroups in leaf veins determines leaf hydraulics, and the transcription levels of most PIP genes increase and decrease through stem development [58].

Plants have trouble absorbing water when they are exposed to abiotic stresses like salt, drought, and low temperatures, as well as ion stress and environmental temperature fluctuations. Plants are able to preserve their water balance through the expression of AQPs being altered by the signal molecules produced by water loss. The majority of the *PeuAQP* genes may be implicated in abiotic stress responses in different organs and tissues, according to an analysis of the expression patterns of the *PeuAQP* family genes under drought, salt, and hormone stress conditions. The gene expression of six *PeuAQPs* is both up- and down-regulated in response to hormone, drought, and salt stress. This suggests that these genes may be important novel genes present in *P. euphratica’*s response to these three stresses and that they might play a progressive regulatory part in the molecular controlling pathways that enrich the abiotic stress compliance of *P. euphratica*. The study of *PeuAQPs’* promoter cis-elements revealed the presence of drought-responsive elements in addition to those linked to hormone regulation in plants, such as gibberellin, abscisic acid, and auxin (as illustrated in Figure 5). Through active oxygen clearance and signal transduction pathways, gibberellin (GA), a crucial phytohormone, helps plants respond to osmotic stress. It was hypothesized that GA controls plants’ osmotic pressure by lowering leaf transpiration because its production and breakdown during the initial phases of osmotic stress modify a plant’s stomatal opening and effect the level of transpiration [59]. Plants produce ABA and respond to osmotic stress to control the guard cells’ plasma membrane ion channels. This results in the long-standing outflow of K+ and undesirable ions, which shrinks the guard cells and closes their stomata [60]. Through the control of gene expression in response to osmotic stress and drought, the ABA response element (ABRE) improves plant tolerance [61]. Moreover, the DELLA (GAI/RGA/RGL1/RGL2/RGL3) protein’s critical node may work in tandem with ABA and GA signals to control the plant response to osmotic stress [62]. The young, rapidly growing plants have the majority of auxin in their tissues. Auxin may stimulate the production of *PeuAQP* genes, which suggests that AQP genes may be involved in the auxin signal system. AQP genes were primarily expressed in the developing tissues of *Populus*. Gibberellin controls the growth of flower organs, stem and root elongation, the germination of seeds, and embryonic development. Genes known as AQP targets are found downstream of the GA signaling cascade. Gibberellin significantly reduced or increased the expression of the *PeuAQP* gene family (Figure 6). Salicylic acid, MeJA, and abscisic acid responsive elements are also present in the *PeuAQPs* promoter cis-acting element. Salt stress caused ABA to be consistently increased in cotton, suggesting that it may be useful in reducing that kind of stress [63]. Additionally, the mechanism of salt tolerance in cotton was supported by the buildup of ABA [64]. MeJA is essential for preventing damage from other elements, heavy metal stress, cold stress, osmotic stress, and salt stress. Stressed plants respond better to MeJA application; they accumulate active chemicals, change physiologically and biochemically, and their endogenous hormone levels rise [65]. Plants’ ability to withstand salt can also be enhanced by the application of salicylic acid [66]. In this study, SA, IAA, GA3, MeJA, and ABA were found to either activate or inhibit *PeuAQPs* (Figure 6), suggesting that AQPs might play a significant role in the intricate network of plant hormone signal transduction. 

The expression patterns of genes in altered tissues or organs designate the probable biological tasks of these genes. The study used qRT-PCR analysis to examine the expression of AQP in *P. euphratica* leaves, stems, and roots during various drought stress periods. The results demonstrated that the *PeuAQP* genes’ relative expression decreased substantially under drought stress conditions. Previous studies demonstrated that AQP gene expression patterns in *A. thaliana* during drought stress varied significantly; for instance, *AtPIP2;1* and *AtPIP2;2* expression were down-regulated [67], although *AtPIP1;3* expression was also seen, and *AtPIPs* expression was up-regulated in certain groups [68]. When *OsPIP1* expression in rice was analyzed under drought stress conditions, Liu et al. [69] found that while all *OsPIP2* gene expression was down-regulated, the expression of these two genes was up-regulated. These findings show that there are complicated patterns of AQP gene expression in plants that are stressed by drought. Comparable to the previously mentioned outcomes, our findings showed that genes like *PeuAQP12* (PIP2;4), *PeuAQP13* (PIP2;5), *PeuAQP14* (PIP2;7), and *PeuAQP15* (PIP2;8) were up-regulated in roots, with mild to moderate drought conditions inducing the most obvious response compared to CK (the control). However, responses to severe drought stress were the most sensitive, with peak values present after 10 days of treatment compared to CK (the control) (Figure 7). The *PeuAQPs* were highly expressed in all organs, but the highest absolute expression was in the young leaves and roots of poplar species. 

Consequently, salt stress might be mitigated by *PeuAQPs*. The profitable yield of desert poplar trees is extremely exacerbated by salinity stress, which is one of the most significant environmental constraints. The qRT-PCR results demonstrate that the relative expression of PeuAQPs are up- and down-regulated under salt stress conditions. This study reveals that three *PeuAQP* genes, including *PeuAQP9* (PIP1;2), *PeuAQP13* (PIP2;5), and *PeuAQP15* (PIP2;8), show varying responses to salt stress. *PeuAQP9* (PIP1;2), *PeuAQP13* (PIP2;5), and *PeuAQP15* (PIP2;8) were meaningfully up-regulated after 48 h of NaCl treatment in young leaves at 24 h and 48 h. However, they decreased at 96 h in young stems and roots. The expression levels of other AQP genes varied, with *PeuAQP12* (PIP2;4) and *PeuAQP15* (PIP2;8) up-regulated in roots (Figure 8). The results suggest that these genes may respond to NaCl stress. Drought, salt, or hormone pressures had an impact on the expression of *PeuAQP* genes as well as cis-acting elements in the promoters of those genes that respond to stress hormones and other stresses, suggesting that *PeuAQPs* have roles in stressful conditions. To sum up, *PeuAQPs* might play a role in PHs, signal transduction, growth and development, and responses to abiotic stressors. The verification of gene function is still the focus of research, not the actual application. Therefore, in order to increase desert poplar stress resistance through molecular breeding, particularly in arid regions, it is imperative to methodically unravel the functions, mechanisms, and molecular regulatory networks of the AQP gene family in response to abiotic stresses. 

## 4. Materials and Methods

### 4.1. A Comprehensive and Sequence Analysis of PeuAQPs

Euphrates poplar AQP sequences were recovered from the National Center for Biotechnology Information (NCBI) databank (www.ncbi.nlm.nih.gov/protein, accessed on 19 April 2024) by entering as a keyword search “aquaporin or MIP and *Populus euphratica*”). To ascertain the AQP genes in *Populus euphratica* (PeuAQPs), the putative 35 AQP proteins in *Arabidopsis* (*AtAQPs*) were employed as the query sequences alongside the whole protein sequence of *P. euphratica* using the BLASTP tool software (https://blast.ncbi.nlm.nih.gov/Blast.cgi?PROGRAM=blastp&PAGE_TYPE=BlastSearch&LINK_LOC=blasthome, accessed on 19 April 2024), and the putative sequences of *P. trichocarpa* were acquired via BLASTP in phytozome at https://phytozome-next.jgi.doe.gov/blast-search (accessed on 19 April 2024). Next, the extracted sequences were retrieved via the Conserved Domains Database (CDD) at (www.ncbi.nlm.nih.gov/Structure/cdd/wrpsb.cgi) (accessed on 19 April 2024) and Pfam. The AQP domain (PF00230) was then employed to search the *P. euphratica* protein database to identify possible alignments via the online HMMER database at https://www.ebi.ac.uk/interpro/ (accessed on 19 April 2024). Lastly, short sequences (less than 150 amino acids in length) were removed from the list. The retrieved sequences were evaluated via the Expasy website and their physicochemical properties, like molecular weight (MW), isoelectric point (pI), instability index, and GRAVY index were projected using the ProtParam tool length. 

### 4.2. Investigating Phylogeny and Motif of PeuAQPs

A phylogenetic tree was built by the whole protein sequences of the PeuAQP gene family members and their orthologous from *P. trichocarpa* and *A. thaliana* plants to investigate the relationships of AQP proteins in *P. euphratica*. *A. thaliana’*s AQP genes were obtained using an online database (https://www.arabidopsis.org/, accessed on 19 April 2024). Using the ClustalW program and the online resource Clustal Omega, the sequence of AQP proteins was aligned [70]. Then, a network tree of AQPs was constructed using MEGA with full-length of protein sequences generated using the maximum likelihood (ML) method with 1000 bootstrap replications through the IQ tree website [71]. The iTOL tool (v6) [72] was used to draw the phylogenetic tree. Moreover, ten conserved motifs in PeuAQPs were retrieved via the MEME motif finder tool (v 5.5.7) set to the default setting [73]. 

### 4.3. Determining the Duplication Genes and Estimating Ka and Ks Values

The duplication procedures amongst *PeuAQP* genes were explored based on comparison, identifying more than 80% midst pairs of *PeuAQP* genes. Likewise, synonymous (Ks) and non-synonymous (Ka) values at each site amongst pairs of replicated genes were calculated via the KaKs_Calculator 3.0 software for Ka and Ks calculation. and their ratio of duplicated genes in the family. The Ka/Ks ratio was likely used at the time to categorize how evolutionary pressure affected the functionality of duplicated genes. The division time of pairs of replicated *PeuAQP* genes were assessed using the synonymous mutation rate of substitution λ per synonymous site per year, as T = (Ks/2λ (λ = 6.5 × 10^−9^)) × 10^−6^ [48].

### 4.4. Prediction of Phosphorylation Site in PeuAQP Proteins

One of the most noteworthy kinds of post-translational modification is phosphorylation. Proteins contain serine, tyrosine, and threonine residues, which are the sites of phosphorylation. These sites play a significant part in response to abiotic stress in plants [57]. The potential phosphorylation regions of *PeuAQP* proteins were projected through the NetPhos 3-1 site via an online tool (https://services.healthtech.dtu.dk/services/NetPhos-3.1/, accessed on April 2024) with a probable value higher than 0.80 [74]. 

### 4.5. Prediction of 3D Structures and Pocket Sites and Interaction Network of PeuAQP Proteins

The three-dimensional (3D) structures of *PeuAQP* proteins were projected via the Phyre2 database [75]. Proteins were aligned using hidden Markov models via HMM-HMM, with the intensive mode designated to improve alignment precision. The validity of the predicted protein model was assessed through Ramachandran plot analysis [76]. The analysis of ligand binding regions (pocket sites) in the anticipated protein models was also completed using the Phyre investigator tool of the Phyre2 server. The 3D protein model was built with a 100% confidence interval for all the positively predicted genes, and the proportions of residue coverage varied from 78 to 98%. Moreover, we created a protein–protein interaction network of 22 PeuAQP proteins based on their homologs in *P. euphratica* versus *A. thaliana* using the String online database (https://string-db.org, accessed on 24 May 2024).

### 4.6. PeuAQP Gene Promoter Analysis

To probe the cis-acting elements in the promoter region, a set of 1500 bp nucleotides upstream of the *PeuAQP* genes were examined as the promoter region. The sequence of the promoter region was explored in order to distinguish the putative cis-regulatory elements via the PlantCARE database [77]. Lastly, the primary cis-regulatory elements were clustered based on their activities. 

### 4.7. Analyses of Synteny in PeuAQPs

The physical site of the *PeuAQP* genes in the *P. euphratica* genome database was evaluated by Tbtools. The MCScanX program (University of Georgia, Athens, GA, USA) was used with defaulting settings to analyze the *PeuAQP* gene duplication occurrences. An analysis was conducted using the dual synteny plotter of Tbtools to determine the homology of AQP genes amongst *P. euphratica* and the other species (*A. thaliana*, *P. trichocarpa*, *P. deltoides WV94-445_v2.0*, and *P. alba* × *P. tremule*\*Populusalba HAP1_717_v5.0*). 

### 4.8. Plant Materials and Treatments

In a greenhouse, two-year-old *P. euphratica* seedlings were cultivated in plastic pots with a soil mixture of Inner Mongolian sand, loamy soil, and peat soil (2:2:1, *v*/*v*). China’s Banner, Inner Mongolia, and Alxa regions were the sources of the seedlings. Three or four seedlings were grown in each pot [52]. Following a period of three to five months of cultivation under carefully regulated conditions (16 h light/8 h dark; 24 h/0 h day/night at 28 °C; and relative humidity ranging from 40–60%, recorded daily) in the greenhouse, four of twelve potted seedlings of identical growth were selected for every treatment. For two weeks, we irrigated the young seedlings with 200 mM NaCl to induce salt stress. After that, we applied the same NaCl doses. The samples were taken at 24 h, 48 h, and 96 h following treatments, as well as at 0 h (untreated; control). Amounts of 100 μM of GA3, 200 μM of ABA, 100 mg/L of IAA, 100 of μM SA, and 100 of μM MeJA were applied in that order to the chosen seedlings to induce hormone stress. An aqueous solution composed of 0.02% (*v*/*v*) Triton X-100 and 0.1% anhydrous ethanol served as the control treatment. Subsequently, we sprayed the entire plant uniformly until the leaves were almost wet. Three leaves of each of the thirty-six seedlings were then collected at 0, 1, 2, 4, 8, and 24 h following treatment. The technique described by Tang et al. [40] was used to treat seedlings during drought stress. Samples (0.2 g) of leaves, stems, and roots were taken from three plants with the same maturity level for both salt and drought stress. Upon collection, every sample was promptly frozen in liquid nitrogen and kept in an ultra-low temperature refrigerator at −80 °C for storage. 

### 4.9. Analysis of AQPs Gene Expression in Desert Poplar under Drought, Salt, and Hormone Stress by qRT-PCR

Using an RNeasy Mini Kit (Hilden, Germany), whole RNA was extracted from the plant materials, following the manufacturer’s recommendations. Using a FastKing gDNA Dispelling RT SuperMIx kit (TIANGEN BIOTECH, Beijing, China) and a previously published technique, the process and reaction system of RT-qPCR were used to create the first-strand cDNA from the total RNA. The A260/A280 ratio was used to measure RNA quality using a TGem spectrophotometer Plus (TIANGEN BIOTECH, Beijing, China). The pairs of precise primers were designed using an online tool of NCBI Primer Blast (https://www.ncbi.nlm.nih.gov/tools/primer-blast/, accessed on 29 April 2024). Supplementary Table S5 contains a list of all the specific primers used in this work. The study conducted by Wang et al. [78] served as the groundwork for the collection of P. euphratica reference genes. The Stratagene Mx3000P equipment (Agilent Technologies, CA, USA) was used to perform quantitative real-time PCR to identify the chemical SYBR Green. The established reaction system is as follows: To make 20 μL, 10 μL of 2× SuperReal Premix Plus (Tiangen), 0.6 μL of each forward and reverse primer (10 μM), 1 μL of the diluted cDNA template, 0.2 μL of 50xROX Reference Dye, and lastly, RNase-free ddH_2_O were added until 20 μL was reached. The amplification procedure consisted of 95 °C pre-denaturation for 15 min, 95 °C denaturation for 10 s, 55 °C annealing for 20 s, and extension at 72 °C for 20 s and 40 cycles. The qRT-PCR analysis was performed with *Peuactin* as a standardized internal control reference gene. The 2^−∆∆CT^ method was utilized to determine the relative abundance of templates in every PCR expansion batch. In order to normalize the expressions of the control samples, the biological triplicates were employed and were set to 1 for gene expression analysis. We performed statistical analysis using IBM SPSS (Armonk, NY, USA), and we determined the *p*-value using the LSD test. To analyze gene expression, we used Duncan’s test and one-way ANOVA. Lastly, the TBtool software (Toolbox for Biologists v1.130, University of Science and Technology, Wuhan, China) produced heat maps to display the results after the transformation of log2 algorithm. 

## 5. Conclusions

A total of 22 AQP novel genes were discovered in the *P. euphratica* genome through the genomic investigation. The identified *PeuAQP* genes were grouped into four major groups based on phylogenetic relationships, motif analysis, and gene structure analysis: TIPs, PIPs, SIPs, and NIPs. Gene duplication, chromosome locations, cis-acting elements, subcellular prediction, phosphorylation site analysis, 3D modeling predictions, collinearity, and protein–protein interaction predictions were then used to analyze these genes. Additionally, we carried out an analysis on the expression patterns of six chosen *PeuAQP* (PIPs) genes in various *P. euphratica* tissues. Using quantitative real-time PCR (qRT-PCR), we also employed the gene expression study of *PeuAQP* under various abiotic stress treatments, like salt, drought, and hormone stress.

To sum up, we found that *PeuAQPs* contain stress response elements and are present in abiotic stress and hormone responses. Expression patterns revealed that certain genes were expressed in leaves, stems, and roots at varying levels. Therefore, *PeuAQP14* and *PeuAQP15* were extremely sensitive to drought stress and *PeuAQP15* to salt stress. The foliar application of PHs activated or inhibited *PeuAQPs*, suggesting that they may mitigate water shortage effects in poplars. This study extends our understanding of AQPs’ functional roles and offers potential for developing drought-, salt-, and hormone-resistant varieties in poplar species. Further research should emphasis the elucidation of the exact molecular mechanisms by which these genes function, the potential interactions with PH signaling pathways, and their application in breeding programs to develop poplar tree species with enhanced resilience to abiotic stresses.

## Figures and Tables

**Table 1:** Ka/Ks analysis and predictable divergence time (MYA).

Gene Pairs	Ka	Ks	Ka/Ks Ratio	Time (MYA)
*PeuAQP11/PeuAQP12*	0.2206796	1.4647707	0.150658	11.4830886
*PeuAQP16/PeuAQP17*	0.4144309	1.5201132	0.272632	20.7798478
*PeuAQP20/PeuAQP22*	0.4754363	NaN	NaN	NaN

**Table 2:** Plant growth and development and phytohormone- and abiotic stress-related cis-elements.

Element	Core Sequence	Function Annotation
ARE	AAACCA	Response to anaerobic treatment
RY-element	CATGCATG	Involved in seed-specific regulation
CAT-box	GCCACT	Regulatory element related to meristem expression
MSA-like	TCAAACGGT	Involved in cell cycle regulation
MBSI	TTTTTACGGTTA	MYB binding site involved in flavonoid biosynthetic genes regulation
ACE	CTAACGTATT	Response to light
O2-site	GATGATGTGG	Response to zein metabolism
ABRE	TACGGTC	Response to abscisic acid
CGTCA-motif	CGTCA	Response to MeJA
ERE	ATTTCAAA	Response to ethylene
G-Box	CACGTT	Response to MeJA
AuxRR-core	GGTCCAT	Response to auxin
TATC-box	TATCCCA	Response to gibberellin
TCA-element	GAGAAGAATA	Response to salicylic acid
TGACG-motif	TGACG	Response to MeJA
W-Box	TTGACC	WRKY binding site involved in abiotic stress responsiveness
HSE	AAAAAATTTC	Response to heat stress
MBS	TAACTG	MYB binding site involved in drought-inducibility
LTR	CCGAAA	Response to low-temperature stress
DRE	TACCGACAT	Response to dehydration, low-temperature, and salt stresses
TC-rich repeats	ATTCTCTAAC	Involve in defense and stress responsiveness
WUN-motif	AAATTTCCT	Response to wounding

## Data Availability

All data supporting the findings of this study are available within the paper and within its Supplementary Materials published online.

## Supplementary Materials

The following supporting information can be downloaded at: https://www.mdpi.com/article/10.3390/ijms251810185/s1.
